# Supplementary material for: Prevalence and characteristics of childfree adults in Michigan (USA)
Source: PLoS One. 2021 Jun 16;16(6):e0252528. doi: 10.1371/journal.pone.0252528 (PMC8208578; doi:10.1371/journal.pone.0252528)
Supplement: Attachment — Submitted filename: rebuttal letter.pdf [file pone.0252528.s001.pdf]

Summary of revisions to:
"Prevalence and characteristics of childfree adults in Michigan (USA)"
(PONE-D-21-11425)

Dear Dr. Shah Md. Atiqul Haq,

We are pleased to submit a revision of our manuscript to PLoS One. We appreciate the thorough and thoughtful feedback from the three reviewers of our original manuscript and believe the new version of the manuscript is much improved as a result. In this letter, we have included your comments and the reviewers' comments followed by an explanation of our response in the revision.

1 Responses to Editor

1.1 "Thank you for sending your article for consideration. It is important and interesting. There is also an opportunity to expand the research by considering others who are with a child."

Our analysis compares *childfree* individuals to *parents* (i.e. individuals who are with a child) as well as to *childless* individuals (i.e. individuals who want a child but cannot have one) and *not-yet-parents* (i.e. individuals who plan to have children in the future). We believe these four groups exhaustively capture all possible parental statuses.

1.2 "Please ensure that your manuscript meets PLOS ONE's style requirements, including those for file naming."

We have prepared the revised manuscript following the PLoS LaTeX template.

1.3 "Please review your reference list to ensure that it is complete and correct."

We have reviewed the reference list to ensure it is complete and that all references are correctly formatted.

1.4 "Please modify the title to ensure that it is meeting PLOS' guidelines. In particular, the title should be "specific, descriptive, concise, and comprehensible to readers outside the field" and in this case it is not informative and specific about your study's scope and methodology."

We have revised the title as: "Prevalence and characteristics of childfree adults in Michigan (USA)"

2 Responses to Reviewer 1

2.1 "...a caveat that merits attention is the very low R^2 in all of the models, ranging from 0.04 to 0.13 in the full models (and even lower in their reduced models). This means that, despite their statistical significance, an enormous amount of variation in life satisfaction, political ideology, and warmth toward childfree individuals are going unexplained by the childfree status. This might suggest that being childfree by itself is not as important as the study believes it to be, that the models may require additional variables, and/or merits some commentary from the authors."

The R^2 values are typical of social science studies of life satisfaction and personality (e.g., Diener et al., 1999; Pinquart et al., 2008). Although larger R^2 values might be desirable if our goal was to explain variation in these dependent variables, our goal was specifically to determine whether parental status in explains any of this variation. We find that in the case of life satisfaction and personality it does not, and in the case of ideology and warmth it does. Adding additional covariates to these models might help us identify *other* variables that explain these dependent variables, but this was not the goal of the paper. We completely agree with you "being childfree by itself is not...important" for explaining some of these dependent variables. Indeed, this is a central finding of our study, that childfree individuals often do not differ from individuals with other parental statuses.

2.2 "Another caveat that merits some attention is the fact that the one survey the authors based their study on was conducted during COVID-19. There thus appears to be some disconnection between the set-up (the literature review, whose research was done in pre-COVID-19 times) and the study (strictly during COVID-19). The authors could amend this connection by better framing the article with respect to COVID-19 and by citing more of the emerging literature on family dynamics amid COVID-19, or by analyzing longitudinal data from before the COVID-19 pandemic (to assess what things were like in the era that the article's cited research are based."

To the best of our knowledge this is among the first studies to explicitly distinguish childfree individuals from those with other parental statuses, and among the first to do so in a representative sample of both men and women. Therefore, relevant pre-COVID data or literature does not exist.

In our discussion of limitations, we now discuss the possibility that our findings may be driven in part by the COVID-19 pandemic. We also identify as a future direction to collect similar post-COVID-19 data to examine potential pandemic influences.

2.3 "Please be mindful of spelling and grammatical errors that surface in the abstract and text."

We have carefully proofread the text of our abstract manuscript for spelling and grammar errors.

2.4 “The authors might consider placing their article in discussion with the following articles, given their research on family dynamics in COVID-19: Lebow, J. L. (2020). Family in the age of COVID-19. Family process & Power, K. (2020). The COVID-19 pandemic has increased the care burden of women and families. Sustainability: Science, Practice and Policy, 16(1), 67-73.”

Thank you for suggesting these additional articles. We have included them in our discussion of the possibility that the COVID-19 pandemic may have influenced our findings.

3 Responses to Reviewer 2

3.1 “The study presents a granular overview of the childfree demographic by disaggregating non-parents into individuals who are childless, not-yet-parents, and finally those who have chosen to be childfree. This was achieved with a simple but elegant set of three questions baked into the survey. In my view, this representative survey, albeit restricted to a single region in the US, is the key contribution of the study. It shows that this specific cohort seems to account to a quarter of the US population.”

We are pleased that the reviewer saw the strength and contribution of our representative sample to the literature.

3.2 “The study is methodologically sound, but the authors could have further advanced the contributions of their study with more sophisticated modeling. The actual analyses performed in the paper are rather exploratory and restricted to estimating a series of ordinary least squares multiple regression.But that doesn’t detract from the paper and the presentation of what is seemingly a representative survey of childfree individuals and their counterparts. My recommendation for future research is to go back to the analysis and further explore the data. I can think of a number of questions that could be probed by deploying further survey waves to collect longitudinal data, and I’m sure the author(s) are similarly making inroads in this direction.”

We appreciate these suggestions and do indeed plan to continue analyzing these data. The *State of the State Survey* is conducted bi-annually, but does not use a panel sample. So, while it may be possible to collect similar data in the future (which we hope to do), it is unfortunately not possible to collect these data from the same respondents. However, we do identify longitudinal studies, and especially within-person and life-course studies, as future directions for research in this population.

3.3 “The paper is immaculately written despite the occasional typo (e.g., “but must lower than”).”

Thank you! We are glad that you found our paper to be clearly written. In the revision, we have carefully proofread the paper to eliminate typographical and grammatical errors including the one mentioned here.

4 Responses to Reviewer 3

4.1 "This is a straightforward article which provides a thorough analysis of 'childfree' individuals in one US state."

Thank you! We are pleased that you found our article to be straightforward and to provide a thorough analysis of the childfree individuals in Michigan.

4.2 "Explicate that the National party in New Zealand is the conservative party."

The sentence now reads: "Adopting a narrower focus, additional work similarly finds that support for taking actions to mitigate climate change translated into support for the center-left New Zealand Labour party, and opposition to the center-right National party, but only for parents."

4.3 "The claim that suspicion of vaccination is "conservative" seems to be overgeneralising from an idiosyncratic feature of the current American political scene. Is it true, for example, that in France, those who are skeptical of vaccines are more right-wing?"

We have clarified that the conservative view is not about suspicion of vaccination in general, but specifically the view that vaccination should be a parent's choice. We have revised the sentence as "This association between parenthood and conservative views has been demonstrated for specific issues as well: in the United States, parents were 15% more likely than non-parents, and very conservative individuals were 230% more likely than moderates, to believe that "vaccines should be a parent's choice"."

4.4 "There is a typographical error in line 362."

We have corrected the typographical error in the revision. The text now reads "...*much* lower than rates of partnership among parents".

4.5 "Reference 4 omits necessary bibliographic information and the reference to the "Times" in the text is ambiguous—specify NYT, Times of London, or whatever."

The article referenced was published in Time magazine. We have corrected the text which originally read "Times magazine". We have also corrected the reference entry to correctly list all bibliographic information.
